# Addressing the Evolution of Cardenolide Formation in Iridoid-Synthesizing Plants: Site-Directed Mutagenesis of PRISEs (Progesterone-5β-Reductase/Iridoid Synthase-like Enzymes) of Plantago Species

**DOI:** 10.3390/molecules29235788

**Published:** 2024-12-07

**Authors:** Maja Dorfner, Jan Klein, Katharina Senkleiter, Harald Lanig, Wolfgang Kreis, Jennifer Munkert

**Affiliations:** 1 Friedrich-Alexander-Universität Erlangen-Nürnberg, Department of Biology, Pharmaceutical Biology, Staudtstraße 5, 91058 Erlangen, Germany; maja.dorfner@fau.de (M.D.); katharina.senkleiter@fau.de (K.S.); wolfgang.kreis@fau.de (W.K.); 2 Department of Plant Physiology, Matthias Schleiden Institute for Genetics, Bioinformatics and Molecular Botany Friedrich-Schiller-Universität Jena, Dornburger Str. 159, 07743 Jena, Germany; jan.klein@uni-jena.de; 3 National High Performance Computing Center (NHR@FAU), Friedrich-Alexander-Universität Erlangen-Nürnberg, Martensstraße 1, 91058 Erlangen, Germany; harald.lanig@fau.de

**Keywords:** *Plantago lanceolata*, *Plantago media*, iridoid biosynthesis, progesterone, PRISEs, cardenolides, site-directed mutagenesis, structural analysis

## Abstract

Enzymes capable of processing a variety of compounds enable plants to adapt to diverse environmental conditions. PRISEs (progesterone-5β-reductase/iridoid synthase-like enzymes), examples of such substrate-promiscuous enzymes, are involved in iridoid and cardenolide pathways and demonstrate notable substrate promiscuity by reducing the activated C=C double bonds of plant-borne and exogenous 1,4-enones. In this study, we identified PRISE genes in *Plantago media* (*PmdP5βR1*) and *Plantago lanceolata* (*PlP5βR1*), and the corresponding enzymes were determined to share a sequence identity of 95%. Despite the high sequence identity, recombinant expressed *Pmd*P5βR1 was 70 times more efficient than *Pl*P5βR1 for converting progesterone. In order to investigate the underlying reasons for this significant discrepancy, we focused on specific residues located near the substrate-binding pocket and adjacent to the conserved phenylalanine “clamp”. This clamp describes two phenylalanines influencing substrate preferences by facilitating the binding of smaller substrates, such as 2-cyclohexen-1-one, while hindering larger ones, such as progesterone. Using structural analysis based on templates PDB ID: 5MLH and 6GSD from PRISE of *Plantago major*, along with in silico docking, we identified positions 156 and 346 as hot spots. In *Pl*P5βR1 amino acid residues, A156 and F346 seem to be responsible for the diminished ability to reduce progesterone. Moreover, the double mutant *Pl*P5βR_F156L_A346L, which contains the corresponding amino acids from *Pmd*P5βR1, showed a 15-fold increase in progesterone 5β-reduction. Notably, this modification did not significantly alter the enzyme’s ability to convert other substrates, such as 8-oxogeranial, 2-cyclohexen-1-one, and methyl vinyl ketone. Hence, a rational enzyme design by reducing the number of hotspots selectively, specifically improved the substrate preference of *Pl*P5βR1 for progesterone.

## 1. Introduction

**Figure 1:**
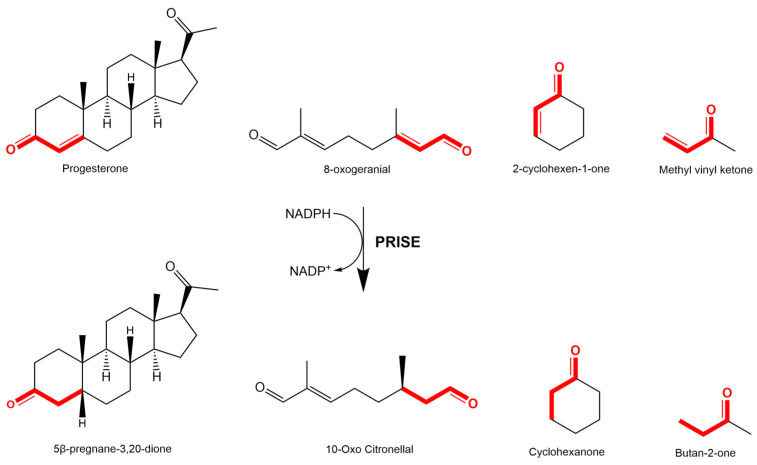
Progesterone, 8-oxogeranial, 2-cyclohexen-1-one, and methyl vinyl ketone (MVK) used as substrates in PRISE reactions. Specific reduction in activated C=C double bonds highlighted in bold red.

The genus *Digitalis* seems to be an exception in the Plantaginaceae family as members from this genus are able to synthesize 5β-cardenolides. In contrast, even close relatives, such as *Erinus* or *Plantago*, have not been shown to accumulate these compounds. On the other hand, iridoids are widely found among Lamiales and are present in those close relatives of *Digitalis*. As far as drug discovery is concerned, both iridoids (e.g., [1]) and 5β-cardenolides are regarded as promising natural compounds [2]. Interestingly, a central step in iridoid as well as in 5β-cardenolide biosynthesis is catalyzed by enzymes now classified as PRISEs (**p**rogesterone-5β-**r**eductase (P5βR)/**i**ridoid **s**ynthase (ISY)-like **e**nz**y**mes), a subfamily among the short-chain dehydrogenase/reductase (SDR) superfamily [3,4,5,6,7]. The overarching term PRISE is used due to the existence of shared conserved motifs, similarities in their structure and sequence, and the same reductive 1,4-addition mechanism (Figure 1) [5,6,7]. In general, PRISEs appear to not only be present in Lamiales but are also commonly found in angiosperms [5,6,8].

The differentiation between iridoid synthase-like enzymes (ISY—converting 8-oxo-geranial into nepetalactol) and progesterone-5β-reductase-like enzymes (P5βR—converting progesterone into 5β-pregnane-3,20-dione; Figure 1) is used to highlight their role in distinct biosynthetic pathways. For instance, the term ISY is used for the enzyme in plants such as *Catharanthus roseus*, which contains iridoids but no cardenolides. On the other hand, the PRISEs of *Digitalis lanata* are usually dubbed P5βR as these plants contain cardenolides but lack iridoids. However, PRISE homologs have also been found in plants producing neither iridoids nor cardenolides, e.g., *Arabidopsis thaliana* L. (*A. thaliana*), and they were therefore sometimes termed steroid reductases (StR; [9]) or were more generally referred to as 1,4-enone reductases [10,11,12].

**Figure 2:**
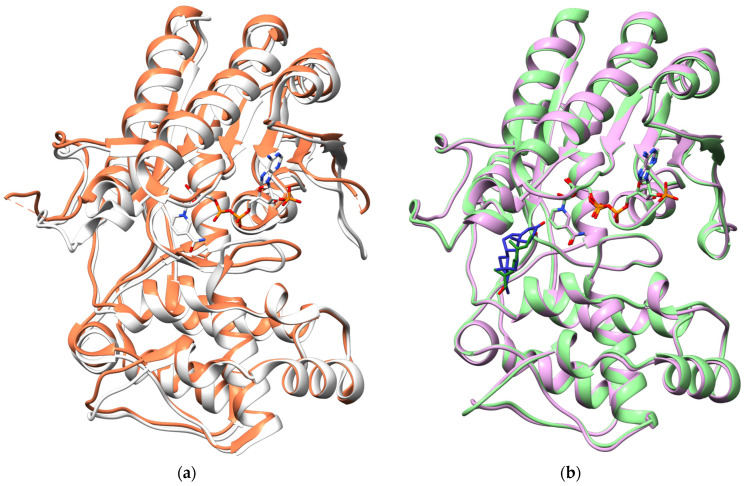
Superimposed crystal structures: (**a**) *Dl*P5βR1 2V6F (orange) and 2V6G (white) with NADP(H) co-crystallized; (**b**) *Pm*MOR 6GSD (violet) including progesterone (blue), 5MLH (green), and 8-Oxogeranial (dark green). These figures were generated using UCSF Chimera 1.17.1.

**Figure 3:**
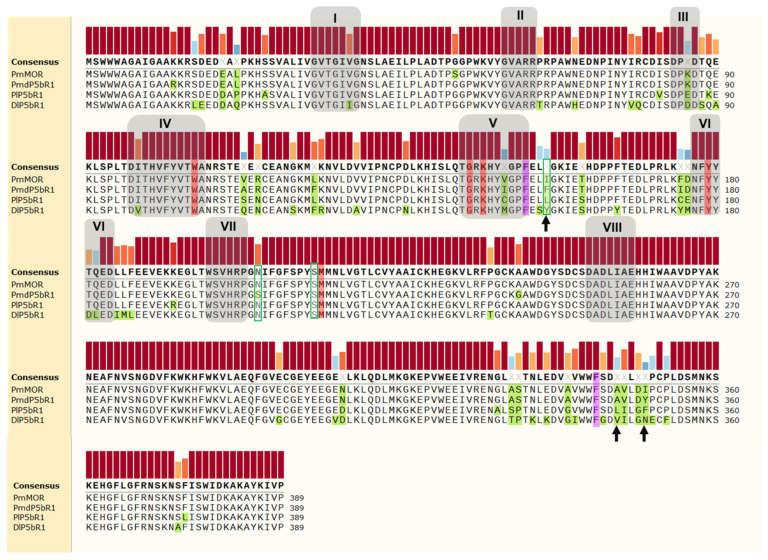
Alignment of the PRISESs from *P. lanceolata*, *P. media*, and *P. major* aligned with their homologs from *Digitalis lanata* using Clustal Omega [27]. The main differences in the amino acid sequences are highlighted in green. The conserved motifs defined by Thorn et al. [3] and by Pérez-Bermúdez et al. [13] are highlighted in gray. The structurally conserved amino acids of the binding pocket identified by Bauer et al. [10] are highlighted in red. The “gatekeepers” F153 and F343 are highlighted in pink [6]. The hot spot amino acid positions identified by Bauer et al. [29] are framed in green. The black arrows mark promising spots for SDM.

Eight motifs were identified to categorize a SDR as a PRISE [3,13]. Motifs I–III including the Rossmann-fold are common among all NADPH-dependent SDRs (Figure 2 and Figure 3). Within the PRISE subfamily, the two catalytically important residues tyrosine and lysine are located in motifs V and VI, respectively, diverging from the standard SDR where a catalytic triad is composed of serine, tyrosine, and lysine [14].

The reaction catalyzed by PRISE (in the presence of NADPH + H^+^) is the reduction of carbonyl-activated C=C double bonds. PRISEs have a relaxed substrate specificity and can accept a wide range of compounds with an alkene conjugated to the carbonyl group of a ketone, e.g., α,β-unsaturated enones in addition to 8-oxogeranial and progesterone (Figure 1) [7,11,15]. One example is the small α-substituted enone methyl vinyl ketone (MVK), a metabolite associated with stress in plants [16]. The ability of PRISEs to reduce this particular compound contributes to the assumption that they have a part in the elimination of reactive electrophile species (RES), which could explain their ubiquitous occurrence in the plant kingdom [17,18].

This assumption is further supported by the fact that multiple genes encoding PRISEs can be found within a single genome and paralogs can be categorized into distinct clusters (Figure S1) [5]. Enzymes of an individual cluster tend to have a preference toward larger substrates such as progesterone or smaller substrates such as MVK or 8-oxogeranial [5,7,8,17,18]. One very prominent substrate-distinguishing feature, which was first described by Petersen et al. [6], is the “phenylalanine clamp” that is located on the outer rim of the substrate-binding pocket and acts as a substrate gatekeeper. This “clamp” modulates the accessibility and the conversion rates of large lipophilic molecules, such as progesterone. PRISEs without the “phenylalanine clamp” discriminate against small hydrophilic molecules, such as MVK and 2-cyclohexen-1-one, and favor progesterone [5,6].

Further analysis is needed to understand the evolution and the physiological functions of PRISEs. Comparing their active sites including the periphery of the very catalytic center can help address the matter. Thus far, most of the research on the substrate preferences of PRISEs and the muteins thereof is conducted in (1) the cardenolide-containing genus *Digitalis*, (2) the iridoid-containing genera *Catharanthus*, and more recently also in *Plantago*, and (3) in the cardenolide- and iridoid-free model plant *Arabidopsis thaliana* (e.g., [4,6,7,10,12,19]).

From an evolutionary point of view, promiscuous enzymes might be driven into specialization under certain conditions [20], and PRISE reactions may be an ancestral biochemical trait in land plants [8]. We focus on PRISEs of *Plantago* species, to investigate the transition in the substrate promiscuity of PRISEs from cardenolide-containing plants to iridoid-containing plants, including stress response reactions.

*Plantago* species are medical plants and are closely related to the *Digitalis* species [21]. Furthermore, crystal structures of the *Plantago major* PRISE *Pm*MOR [19] and *D. lanata* PRISE *Dl*P5βR are also available, contributing to structural analysis with respect to enzyme-substrate promiscuity [3].

In this study, we identified PRISE genes of *P. lanceolata* (*PlP5βR1*) and *P. media* (*PmdP5βR1*). We produced recombinant forms of the encoded enzymes (r*Pl*P5βR1 and r*Pmd*P5βR1) and investigated their protein structures. We examined catalysis-discerning factors between them to explain the discrepancies in their substrate preferences, mainly regarding the conversion of 8-oxogeranial (iridoid pathway metabolite) and progesterone (cardenolide pathway metabolite). Site-directed mutagenesis (SDM) was used to modify their catalytic properties.

## 2. Results and Discussion

### 2.1. Identification and Heterologous Expression of PRISE from P. media and P. lanceolata

Using primers (Table S1) deduced from a PRISE mRNA sequence of *P. major* (GenBank: ADG56541.1), we succeeded in the amplification of 1200 bp PCR products from cDNA of both *P. media* (PmdP5βR; OR269723.1;WKF48833.1) and *P. lanceolata* (PlP5βR; OR269722.1; WKF48834.1). These cDNAs were each cloned into a pDEST17 expression vector via restriction-free cloning [22] and then sequenced and submitted to the PubMed Databank. They contained the complete coding sequences (CDSs), each composed of 1170 bp (389 amino acids). The genes as well as the encoded enzymes showed an overall high sequence similarity to PRISEs from, e.g., *Plantago major* (ADG56541.1), *Digitalis lanata* (AAS76634.1), *Catharanthus roseus* (AIW09143.1), and *Arabidopsis thaliana* (ABU55811.1) (Table 1). The enzymes considered as ISY or P5βR are widespread in the plant kingdom [23,24], and they generally show high sequence similarities, despite supposedly participating in different biosynthetic pathways, as well as being involved in stress reactions [13] or vein-patterning [25]. This resulted in the assumption that PRISEs can be regarded as a catalytic reservoir for specialized metabolisms across land plants [8]. Clustering PRISEs in either ISY or P5βR is not possible since P5βR activity is also common among ISY [5]. PRISEs can be used as taxonomic markers [26] hence it is perspicuous that the *Plantago* PRISEs are more related to the *Digitalis* PRISE (both genera belong to the Plantaginaceae family) than to the respective *Arabidospis* (Brassicaceae) or *Catharanthus* (Apocynaceae) enzymes/genes (Table 1). *Pl*P5βR and *Pm*dP5βR have an amino acid sequence identity of 92.8%, differing by only 26 residues and exhibiting no gaps. Based on the phylogenetic analysis of Schmidt et al. [7], both were found to fit into the Cluster I category similar to the P5βR from *Plantago major* [7,10] (Figure S1). Database research and nucleotide blasts in the available data sets of *Plantago* and *Digitalis* species revealed that more than one PRISE might reside in their genomes. We focused on closely related homologues (Table 1) in order to simplify our approach and identify hot spots that are responsible for substrate discrimination.

### 2.2. Substrates

PRISEs are known to catalyze the reduction of C=C double bonds of α,β-unsaturated carbonyl compounds in the presence of NADPH + H^+^ as a co-substrate. The diversity of accepted substrates became apparent in the studies of Herl et al. [9], Burda et al. [15], and even more comprehensively in Durchschein et al. [11] in which a recombinant form of an *A. thaliana* PRISE (*At*StR) was exposed to various compounds, including small linear and cyclic enones or enals and also larger molecules such as α, β-unsaturated steroids.

We focus on four substrates (Figure 1), namely progesterone (biosynthetic precursor of 5β-cardenolides), 8-oxogeranial (central metabolite of the iridoid pathway), 2-cyclohexen-1-one (cyclic enone regarded as a small mimetic of progesterone), and MVK (a toxic reactive oxygen species, which has an impact on anti-stress and oxidation processes in plants).

Due to the limited availability of 8-oxogeranial, its mimetic citral was used as a substitute by others [19]. We decided to conduct experiments with 8-oxogeranial instead of its mimic and used a published protocol for its synthesis and optimized the methods to improve both yield and reaction time [4]. We accomplished this by incorporating a microwave-assisted step, which reduced the reaction time of step two (Figure S2) from 6 days to 10 min. Microwave-assisted reactions generally proceed with greater selectivity under milder and simpler conditions, minimizing by-product formation and increasing overall yield compared to analogous homogeneous reactions. Our optimization strategies resulted in a protocol that enabled us to synthesize 8-oxogeranial in less than 6 h, with a 30% yield. Synthesized 8-oxogernial was compared to a respective standard compound by GC/MS analysis (Figure S2). The method is provided in the Supplemental Information (Method: Synthesis of 8-oxogeranial).

### 2.3. Substrate Preferences of Wild-Type PRISEs

As mentioned before, the CDSs of the PRISEs from *P. media* and *P. lanceolata* were cloned into a pDEST17 vector and recombinant forms of *Pmd*P5βR1 and *Pl*P5βR1 were expressed in *E. coli* soluBL21. The recombinant proteins, produced after the induction of gene expression with IPTG, were isolated. They contained a 6× His-tag and were purified by Ni-NTA affinity chromatography as described by Bauer et al. [10] (Figure S7). The recombinant forms of the enzymes were termed r*Pmd*P5βR1 and r*Pl*P5βR1 after being assayed with progesterone.

Progesterone is generally tested as a standard substrate in PRISE studies. The enzymes r*Pmd*P5βR and r*Pl*P5βR were examined under conditions established with the wild-type r*Dl*P5βR [28,29]. It was demonstrated that r*Pmd*P5βR (832 nkat mg^−1^) converted progesterone around 70–100 times more efficiently than r*Pl*P5βR (12 nkat mg^−1^; Figure S4). In fact, the r*Pl*P5βR did not even convert progesterone efficiently enough to reliably determine kinetic constants for this compound (Table 2). Kinetic constants of the wild-type PRISEs were determined photometrically for the selected substrates (Figure 1) by measuring the oxidation of the co-substrate of NADPH. The conversion efficiencies were assessed and compared using *k_cat_*/K_M_ ratios calculated for each tested substrate (Table 2) [29].

Interestingly, r*Pmd*P5βR accepted progesterone almost as well as r*Pm*MOR of *P. major* (Table 2) [10,19]. However, r*Pmd*P5βR (0.16 s^−1^) converted progesterone 8 times more efficient than the r*Dl*P5βR1 (0.02 s^−1^), which, in turn, has been proven to be involved in cardenolide formation via gene knock-down experiments [17]. This is somewhat surprising but corroborates with other reports that have shown that PRISEs of non-cardenolide plants convert progesterone more efficiently than their relatives in cardenolide-producing plant species [5]. Hence, an evolutionary trend in shaping PRISEs toward improved progesterone conversion for 5β-cardenolide biosynthesis is not observed. This issue will be addressed in more detail below when discussing the structural features of the enzymes.

Both PRISEs investigated here showed similar turnover rates (*k_cat_*) for 8-oxogeranial, with r*Pmd*P5βR exhibiting a slightly higher affinity toward 8-oxogeranial and also toward other small substrates (Table 2). Investigating a PRISE from *P. major* (*Pm*MOR), Fellows et al. [19] reported efficiencies in a similar range as we observed. Munkert et al. [5] demonstrated that 8-oxogeranial is an excellent substrate for PRISEs of various origins, reaching *k_cat_*/K_M_ ratios of over 10^5^ M^−1^ s^−1^, even in non-iridoid plants such as *Erysimum crepidifolium*. Therefore, the authors suggested that ISY activity (i.e., 1,4-reduction of 8-oxogeranial) is intrinsic to angiosperm PRISEs. This may be explained by the structural similarity of 8-oxogeranial to chemicals that were converted long before iridoid or cardenolide pathways evolved. In this context, it is interesting to note that certain stress-related 1,4-enones regarded as reactive electrophile species (RES) can be detoxified by PRISEs and in turn have an impact on PRISE gene expression. PRISE knock-down can result in metabolic changes in response to, e.g., exogenous MVK [18]. Therefore, mimics of this type of chemicals, MVK and 2-cyclohexen-1-one, were tested here. For both substrates, the turnover rates for r*Pl*P5βR and r*Pmd*P5βR are similar. r*Pl*P5βR exhibited slightly lower affinities toward the substrates with K_M_ values of 100 µM for MVK and 66 µM for 2-cyclohexen-1-one, respectively, whereas r*Pmd*P5βR exhibited much higher affinities with lower K_M_ values of 33 µM and 19 µM for MVK and 2-cyclohexen-1-one, respectively.

Regarding catalytic efficiencies and substrate affinity, 2-cyclohexen-1-one, MVK, and 8-oxogeranial appear to be better substrates than progesterone for both the recombinant PRISEs studied here. This supports the cluster categorization of Munkert et al. [5], who proposed lower preferences for progesterone of the Cluster I PRISEs to which r*Pl*P5βR1 and r*Pmd*P5βR1 belong. However, the difference in progesterone acceptance is surprising due to the high sequence similarity among the *Plantago* PRISEs (Table 1).

### 2.4. Protein Modeling, Substrate Docking, and Structural Analysis

Homology models were constructed to identify residues and structural features that may be responsible for this effect. Proteins with high sequence identity usually have similar structures [30,31]. This also holds true for the currently available crystal structures of various PRISEs (Figure 2). Due to their similarity, we were able to produce reliable models without actually crystallizing both PRISEs.

PRISEs of *P. lanceolata* and *P. media* were modeled on the crystal structures 6GSD and 5MLH reported for *Pm*MOR [19]. Both templates contained the co-substrate NADPH + H^+^ and either co-crystallized progesterone (6GSD) or 8-oxogeranial (5MLH) (Figure S5; Table S4). The sequence identity among the three enzymes was more than 93%. The models were generated using the SwissModel, applying standard protocols [32]. When assessing the quality of the models, we evaluated the Global Model Quality Estimation (GMQE) score. All scores were above 0.9 (Table S4), indicating a high level of accuracy for the models [33]. We subsequently used these models for our molecular docking experiments.

Once the models were established, we identified possible influential residues within a 5 Å radius of the binding pocket by positioning progesterone, 8-oxogeranial, MVK, and 2-cyclohexen-1-one in silico through molecular docking using AutoDock Vina v. 1.2.5 into the corresponding model [34,35] (Figure S5).

We also modeled r*Pl*P5βR1 and r*Pmd*P5βR1 on the available *Digitalis lanata* structure 2V6G. All generated models were similar, which was obvious due to the high structural and sequential similarity among the PRISE enzymes considered here [7]. The resulting empirical binding free energy score represents the binding affinity in [kcal/mol] and was calculated using the Gibbs free binding energy formula, which includes electrostatic interactions, hydrophobic interactions, hydrogen bonding, and the flexibility of rotatable bonds [36]. Only the docking of progesterone yielded significant differences in scores, which is consistent with the high discrepancy in efficiency observed in vitro (Table 3). All other docking scores represent similar binding affinities, fitting the kinetic parameters that were determined for the small substrates, which were all converted with a very similar turnover rate (Table 3).

### 2.5. Identifiying Key Residues Contributing to Substrate Preferences

**Figure 4:**
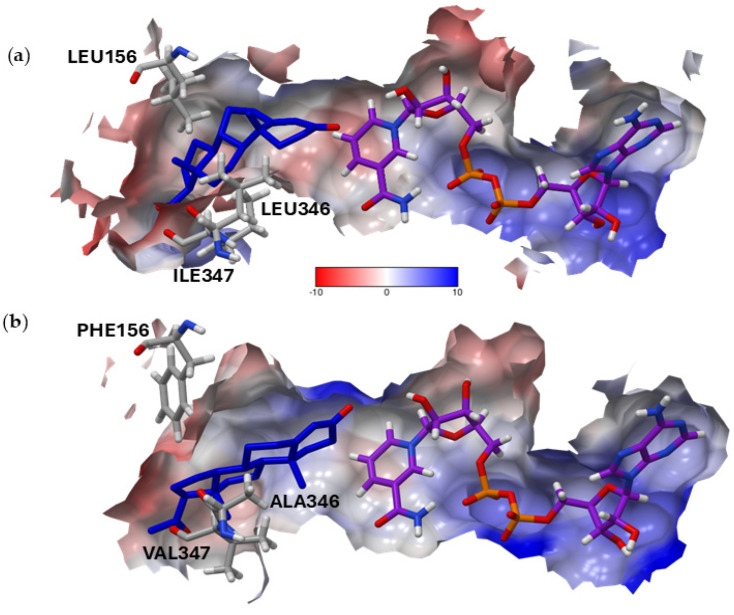
The surface depiction of the binding pocket. Progesterone (blue), NADP (purple), and residues 156, 346, and 347 are shown in stick atomic form. The surface is colored according to its Coulombic surface coloring using UCSF Chimera and a color-coded scale: red (−10 kcal/mol·e), white (0 kcal/mol·e), and blue (10 kcal/mol·e). (**a**) r*Pl*P5βR with leucine 156, leucine 346, and isoleucine 347; (**b**) r*Pmd*P5βR with phenylalanine 156, alanine 346, and valine 347.

Site-directed mutagenesis (SDM) can help to understand the actual dynamic nature of substrate attachment, catalysis, and product release that are not represented by the docking procedure and the docking score. We examined amino acids influencing enzyme activity and cross-checked the different amino acids close to the docked substrate in order to identify the specific amino acids responsible for the difference in progesterone conversion and then mutated them via SDM (Figure 3 and Figure 4).

The direct comparison of the four closely related PRISEs of *D. lanata*, *P. media*, *P. major*, and *P. lanceolata* allowed us to identify 16 positions that did not result in a consensus sequence (Figure 3). We had a more detailed look at these positions and analyzed them based on the findings of [6,7,10,29]. Most discovered amino acids are far off the active site and the conserved motifs, which is why we did not investigate them further in this study.

As suggested by Bauer et al. [10], 17 key residues within a 4.5 Å radius of the docked progesterone were considered important for catalysis. Seven of those are conserved within the PRISEs (Figure 3). Three amino acids at positions 156, 205, and 248 were considered as possible hot spots, which may have an impact on enzymatic activity. Y156, N205, and S248 were considered to be responsible for the low catalytic efficiency of the PRISE of *D. lanata* [29]. Position 248 was not considered further since PRISEs of *P. media* and *P. lanceolata* are sharing the same amino acid (serine) there. Position 205 differed within PRISEs of *P. media* containing serine and *P. lanceolata* containing asparagine (Figure 3). These polar amino acids are amidic or hydroxylic and have low hydropathy scores of −0.8 and −3.5, respectively [37]. However, both amino acids have polar yet uncharged side chains, and an increase in converting progesterone was only observed when mutating this position into more hydrophobic amino acids like alanine (−1.8), methionine (−1.9), or leucine (−3.8) [29]. The same applied to those amino acids when they were occurring naturally [7,29]. Therefore, this hot spot was also not considered to be responsible for the discrepancy in the turnover rate of progesterone (Figure 3). This only left position 156 and its associated amino acid to be investigated.

Within the 7 conserved amino acids, Petersen et al. [6] highlighted 2 phenylalanines (Figure 3) and described a “gatekeeping” structure characterized by these 2 phenylalanines in positions 153 and 343 (“phenylalanine clamp”) in *At*StR, a PRISE of *A. thaliana*. These two phenylalanines are highly conserved in many PRISEs, including the *Plantago* PRISEs investigated here (Figure 4), and seem to be particularly important for substrate discrimination [6,38]. Strangely enough, a tendency toward modifications in these positions is not observed in cardenolide-producing plant species [39] (own protein blast, September 2024).

In our study, we noticed that the angle of the “phenylalanine clamp” depends on the amino acid at position 156. F343 of the “phenylalanine clamp” is part of a rigid helix backbone while F153 is attached to a flexible loop [40]. Due to this flexible attachment, the orientation of F153 may depend on the amino acid at hot spot 156. Therefore, alterations might modify the angle of the “phenylalanine clamp”, which could impact the access of the substrate to the catalytic center. The same might apply to possible angles between the core of an enzyme and a loop conceivably involved in substrate channeling [41]. For example, a PRISE from the iridoid-producing plant *C. roseus* (r*Cr*ISY) exhibited very poor progesterone 5β-reduction [5], which correlates with the narrowed angle of the substrate entrance region (F343 ∡ F153 = 22.6°; in contrast to *Pmd*P5βR1 F343 ∡ F153 = 79°).

The role of the “phenylalanine clamp” was addressed in a series of SDM experiments in various PRISEs [6,38]. Recently a new pair of potential gatekeepers (W109 and W345) was identified [38]. This and other observations resulted in a concept that suggests more than one option for various substrates to reach the catalytic site of certain PRISEs, which can be visualized by highlighting so-called solvent channels (e.g., [42]). When analyzing the open and closed state of a PRISE from *C. roseus* (PDB entry: 5EMH), Sandholu et al. [41] found two channels for substrate entry, a “top-channel” and a “side-channel”, supporting the channel concept when investigating enzyme activities. Smaller dimensions of a channel may simply disable access to the active site for larger molecules such as progesterone. Even so, the substrate specificity of enzymes may be a complex interplay of different residues and they may be steered by the dynamics of the flexible loop regions [41]. However, these loop regions seemed to be very similar in *Pl*P5βR1 and *Pmd*P5βR1 based on our models and might not be responsible for the discrepancy in progesterone conversion, as observed in *Pl*P5βR1 and *Pmd*P5βR1 (Figure 2).

Finally, Petersen et al. [6] demonstrated via MD experiments that the quadruple mutant r*At*StR_F153A_F343A_V156F_V345F, in which the “phenylalanine clamp” is slightly shifted outwards relative to the catalytic center, allowed an increase in progesterone agility while the trapping of smaller molecules became lost.

In conclusion, after similar considerations and a detailed analysis of the literature, we focused our study on only 3 positions, namely 156 (hot spot), 346, and 350, in which *Pl*5βR1 and *Pmd*5βR1 differ. All 3 positions are in close vicinity to the gating phenylalanines 153 and 343. However, position 350 seemed to be too remote from the “phenylalanine clamp” to have an impact. We decided to mutate the amino acid in position 347 as a control, expecting no obvious changes in progesterone conversion.

**Figure 5:**
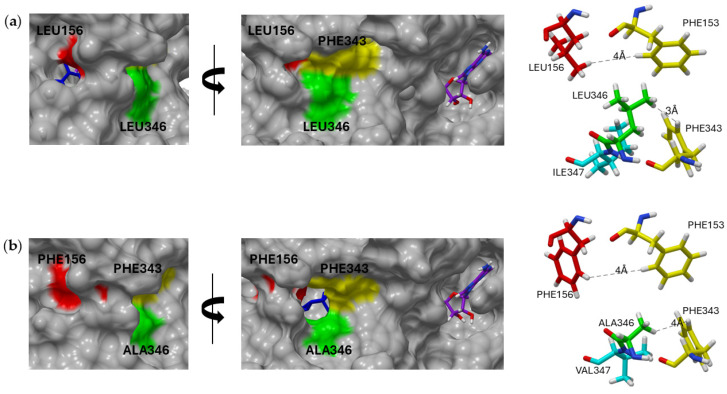
Surface depiction of PRISEs of both *P. lanceolata* and *P. media* with residues 153 (yellow), 156 (red), 343 (yellow), 346 (green), and 347 (cyan) highlighted including progesterone (blue) and NADP (purple). Distances of residue 156 and 346 to “phenylalanine clamp” 153/343 marked in stick depiction of residues: (**a**) *Pl*P5βR with leucine located at both residue positions 156 and 346; (**b**) *Pmd*P5βR containing phenylalanine in 156 and alanine in 346.

Within *Pl*P5βR, both residues (156 and 346) are leucines, whereas in *Pmd*P5βR, phenylalanine and alanine are present in positions 156 and 346, respectively. The surface visualization showed (Figure 5) that the residues were positioned at the rim of the substrate binding pocket. Due to their position and our previous literature study, we considered that these residues possibly assist the gating and therefore could have an impact on progesterone access to the catalytic site (Figure 5).

### 2.6. Modulating Substrate Preferences via Site-Directed Mutagenesis

**Figure 6:**
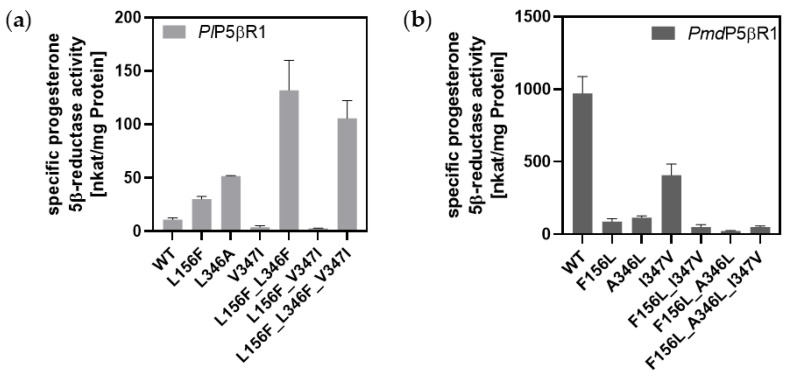
Specific activity of PRISEs from: (**a**) *P. lanceolata* and (**b**) *P. media*. Mutations introduced via site-directed mutagenesis increased progesterone 5β-reductase activity in PRISE of *P. lanceolata*. Progesterone (0.3 mM) was used as substrate and regeneration system with NADP^+^ as co-substrate was conducted with standard P5βR assay and detected via GC-MS.

Our theoretical considerations were assessed via SDM experiments (Figure S8). In the first screening, specific activities for the conversion of progesterone (nkat mg^−1^ protein) were measured. Substituting leucine for phenylalanine-156 in r*Pl*P5βR1 showed a 3-fold increase in progesterone-converting activity when compared to wild-type r*Pl*P5βR1 activities. A 5-fold increase was seen when alanine was substituted for leucine-346 in r*Pl*P5βR1. The double mutant r*Pl*P5βR1_L156F_L346A converted progesterone more than 15 times more efficiently than r*Pl*P5βR1 (Figure 6). r*Pmd*P5βR1 was mutated in a similar manner, which resulted in the muteins *Pmd*P5βR1_F156L, *Pmd*P5βR1_A346L, and *Pmd*P5βR1_F156L_A346L. As predicted, progesterone conversion was affected negatively (Figure 6).

Although a complete catalytic inversion of the enzymes was not achieved, probably due to the fact that progesterone is a large and bulky substrate, these results still demonstrated that single or double mutations in the critical region of the highly conserved gatekeeping phenylalanine 153 and 346 can result in modulations in progesterone conversion.

Substrate specificity differs within the PRISE family. This comes as no surprise looking at the physiological functions of the ubiquitously expressed but separately evolved PRISEs. We have already observed that ISY activity is high within the two PRISEs from *P. lanceolata* and *P. media* and that site-directed mutagenesis in the recombinant PRISEs from *P. lanceolata* and *P. media* altered the conversion of progesterone. In a more detailed analysis, we not only determined kinetic constants and catalytic efficiencies for the single and double mutants of r*Pl*P5βR1 and r*Pmd*P5βR1 with progesterone, but also for 8-oxogeranial, the substrate within iridoid biosynthesis, 2-cyclohexen-1-one, a small mimic of progesterone, and MVK, as a plant stress response marker (Table 4).

The kinetic constants for progesterone supported the GC-MS data that were determined to calculate the specific activity. The photometrically measured catalytic efficacy for progesterone increased around 6 times (6318 s^−1^ M^−1^) in the double mutant r*Pl*P5βR_L346A_L156A compared to the single mutant, mainly due to the higher affinity toward (K_M_ 44 µM) progesterone (Table 4). The single substitution of leucine for the smaller alanine or the larger phenylalanine at positions 156 and 346 in PRISEs from *P. lanceolata* mainly increased the substrate turnover for all tested substrates and in some cases also increased the affinity (Table 2 and Table 4). The catalytic efficacy of the smaller and less hydrophobic substrates 8-oxogeranial, MVK, and 2-cyclohexen-1-one was at least 10 times higher than that of progesterone. For example, the small, cyclic progesterone-mimic 2-cyclohexen-1-one was converted 3 times more efficiently by the double mutant than the original *Pl*P5βR. Interestingly mutations in the PRISEs of *P. media* only influenced the catalytic efficiency for progesterone, MVK, and 2-cyclohexen-1-one but not for the natural substrate 8-oxogeranial, which was accepted best by all other mutants.

Substituting smaller residues on the rim of the binding pocket for larger more hydrophobic amino acids is thought to trap smaller residues within the substrate-binding pocket promoting the enzymatic reaction [29]. The opposite effect can be observed once the “phenylalanine clamp” is missing. For example, one of the PRISE paralogues from *C. roseus* has shown higher affinity toward the larger substrate progesterone compared to the smaller more polar substrate 2-cyclohexen-1-one, which was converted with poor efficiency [5]. Substituting the two phenylalanines 153 and 343 in a PRISE of *A. thaliana* with the smaller, less hydrophobic alanines resulted in an inversion of the efficiency to convert progesterone and 2-cyclohexen-1-one [6]. The discrimination factor expressed as relative activity toward 2-cyclohexen-1-one and progesterone changed from a 15 in favor of 2-cyclohexen-1-one in the wild-type r*At*StR to 274 in favor of progesterone in the r*At*StR_F153A_F343A mutant. The trend was already observed in the respective single mutants [6].

Mutating positions 156 and 346 to resemble the “phenylalanine clamp” to counteract the activity loss caused by its absence did not show the desired effect [6]. This corroborates our findings (Figure 6; Table 4).

By substituting leucine-346 positioned on the periphery of the substrate-binding pocket in r*Pl*P5βR with alanine, the conversion activity and affinity for progesterone as a substrate improved. The mutations in r*Pmd*P5βR were not as impactful as they did not result in a significant change in activity (Figure 6). Kries et al. [40] suggested that the hydrophobic residue I345 in the ISY of *C. roseus* (residue 346 in r*Pl*P5βR and r*Pmd*P5βR) acts as a hinge influencing the conformation of a loop ranging from G150 to D162 (corresponding to G151 to D163 in r*Pl*P5βR and r*Pmd*P5βR) allowing for an either closed or opened binding pocket. A smaller alanine at this position could promote a more open conformation of the binding pocket, potentially improving access for larger substrates. This suggests that this residue influences substrate entry dynamics into the binding pocket rather than directly affecting the catalytic reaction.

In general, new metabolic pathways cannot be designed from scratch, but they may instead emerge from substrate-promiscuous enzymes using bits and pieces from cell metabolism or the environment. Gene duplications may also contribute to this process by providing modified biocatalysts [20]. In this manner, plants can generate an entire range of metabolites, some of which may provide benefits to the producer or the population. PRISEs are an excellent subject for studying enzyme evolution in specialized metabolisms since they seem to have a basic function of trapping and converting and maybe even detoxifying small and hydrophilic molecules, such as MVK. Preserving or even improving this basic reaction mechanism may be beneficial to the conversion of novel substrates. Modifications may occur in the periphery, which is instrumental in shaping substrate entrances, channels, and product exits. Through extensive research (Table S3), including the results of this study, key residues and structures influencing the plasticity of the substrate-binding pocket of the PRISEs were examined and we have revealed the complexity of enzymatic activity relative to a putative substrate.

## 3. Materials and Methods

### 3.1. PRISE Identification and Sequence Analysis

Seeds of *P. lanceolata* were collected from wild populations in July 2016 in Obereuerheim (Bavaria, Germany), while simultaneously seeds of *P. media* were collected from wild populations in Obbach (Bavaria, Germany). The seeds were germinated, and the plants were cultivated in the greenhouse of Pharmaceutical Biology, Friedrich-Alexander-University Erlangen. The youngest leaves of the 4-week-old plants were harvested and frozen in liquid nitrogen. Total RNA was isolated using Monarch^®^ TotalRNA Miniprep Kit (New England Biolabs, Ipswich, MA, USA). In total, 500 ng of total RNA was used for cDNA synthesis using the RevertAid H Minus First Strand cDNA Synthesis Kit (Thermo Fisher Scientific Inc., Waltham, MA, USA). The PRISEs were amplified using primers designed against the PRISE of *P. major* [10]. The Clustal Omega was used for the identification of PRISE motifs and the integration of sequences into one of the two known PRISE clusters [27].

### 3.2. Heterologous Expression of PRISE

PRISEs of *P. lanceolata* and *P. media* were cloned into pDEST17 using a restriction-free cloning strategy, which was used for PRISEs successfully before [17,18], using primers (Table S1) designed with rf-cloning.org [43].

Correct integration of *Plantago* CDSs in the expression vector was verified by sequencing (Eurofins Genomics, Ebersberg, Germany) using standard primers against T7 promoter and T7 terminator. Positive constructs were used for the transformation of *E. coli* SoluBL21. The heterologous expression of *Plantago* PRISEs was realized in 1 L LB medium as described in detail previously [18]. SDS-PAGE and semi-dry immunoblotting (Figure S1) were carried out following QIAexpress Detection and Assay Handbook (QIAgen, Hilden, Germany). We used 12% Bis-Tris polyacrylamide gels for SDS-PAGEs and nitrocellulose membranes for electroblotting. Recombinant PRISEs were detected using mouse anti-His (mixture of RGS-, Tetra-, and Penta-His antibodies; dilution 1:2000; QiAgen, Hilden, Germany) and anti-mouse IgG-peroxidase antibodies (Sigma, Munich, Germany). As a result, we detected recombinant proteins as the chemiluminescence of 3-aminophtalate released from luminol.

### 3.3. Homology Model and Generation of Substrate Structures

The crystal structure of the progesterone 5β-reductase from *P. major* (PDB-ID: 6GSD and 5MLH) served as a template for generating the homology model of *Pl*P5βR and *Pmd*P5βR. Both crystal structures were holoenzymes, 6GSD containing progesterone and NADP^+^, and the template complex 5MLH included co-crystallized 8-oxogeranial and NADP^+^. The sequences of the target protein were generated using the web service SWISS-Model [32] via alignment using BLAST [44] and HHBlits [45] as algorithms (Table S4).

Once the target structure was modeled, the co-substrate was positioned according to the coordinates of the *P. major* model. Each mutation was introduced using the mutate and rotamer functions of UCSF Chimera version 1.17.1 while avoiding any steric collisions [46].

Progesterone, 8-oxogeranial, MVK, and 2-cyclohexen-1-one were generated based on their canonical SMILES spring (Simplified Molecular Input Line Entry Specification), originating from PubChem (National Center from Biotechnology Information, 2020). Prior to the docking experiment, Avogadro version 1.2.0 was used to perform a two-step energetic minimization on all four substrates [47].

All substrates were docked within the binding pocket with AutoDock Vina v. 1.2.5 [35] and the resulting ternary protein complex was visualized with UCSF Chimera version 1.17.1 [46]. The original position of progesterone within the crystal structure of *P. major* (PDB ID: 6GSD) was used as a guideline to determine the active site. The center of this active site was defined as a three-dimensional grid with coordinates X = −31.2, Y = −17.1, and Z = 19.8 and had a size of 20 Å × 20 Å × 20 Å. The docking parameters were set with an energy range of 10 and an exhaustiveness value of 50, which were the default settings applied to all docking experiments. Prior to docking, the structures were minimized using the Amber ff14SB forcefield for standard residues and the Gasteiger method for residues such as ions and small substrates.

### 3.4. SDM of PRISEs

In vitro mutant construction was carried out using PCR in a FlexCyler2 Thermocycler. The primers were designed with OligoCalc [48] (Tables S2 and S3) and supplied by Eurofins Genomics (https://eurofinsgenomics.eu/, accessed on 3 July 2023). The PCR protocol included an initial denaturation step at 98 °C for 30 s, followed by denaturation at 98 °C for 10 s, an annealing step (temperature dependent on the primers) for 30 s, and a final extension at 72 °C for 10 min. After amplification, the PCR product was digested with DpnI to remove template DNA. The DNA was then purified using the Macherey-Nagel NucleoSpin^®^ kit (Macherey-Nagel, GmbH & Co.KG, Düren, Germany). Purity and concentration were assessed using a NanoDrop ND-2000 (PeqLab Biotechnology GmbJ, Erlangen, Germany), with the A_260/280_ absorption ratio used to evaluate nucleic acid purity. The mutations were verified via sequencing through the services of MWR Eurofins. Sequence analysis was conducted using the online tools BLASTP 2.9.0+ [49] and Clustal Omega [27]. We confirmed successful mutations by visualizing the sequences with SnapGene Viewer 5.1.3. 

Once the mutation was confirmed, mutant proteins were expressed in BL21 (DE3) *E. coli*. The plasmid uptake was verified via PCR, using the FastGene^®^ Optima HotStart ReadyMix (NIPPON Genetics Europe GmbH, Düren, Germany).

The His-tag at the N-terminus of the protein allowed for purification by competitively eluting the tagged protein with imidazole (250 mM) via affinity chromatography using a batch-method. The protein concentrations were measured using the Bradford method [50].

### 3.5. Kinetic Data

#### 3.5.1. UV/Vis-Spectrophotometer

The assay was performed according to [5] with purified protein in concentrations ranging between 0.05 and 0.2 mg mL^−1^ and 0.2 mM NADPH in 990 µL of phosphate assay buffer with a pH of 8.0. After 10 µL of substrate was added, the measurements were taken for 300 s upon extinction at 40 °C (λ = 340 nm). The substrates 2-cyclohexen-1-one and MVK were measured within the concentration range of 0.05 mM to 0.4 mM. Progesterone was measured in higher concentrations from 0.1 mM to 0.6 mM. DMSO (40 µL) was added to avoid or stop the precipitation of progesterone in higher concentrations (0.4–0.6 mM). Assays with 8-oxogeranial were performed with lower concentrations from 0.015 mM to 0.2 mM as other P5βR1 had shown high turnover rates toward the substrate.

Once the assay was completed, the recorded progress curve was displayed with the SHIMADZU UVProbe 2.34 Software. The relation to the substrate concentration and reaction rate was analyzed using the Michaelis–Menten equation and resulted in the enzymatic kinetics constants V_max_ and K_M_. The parameters were linearized in the Hanes plot relating [S] (x-axis) against [S]/v (y-axis). The graphs, catalytic constants (*k_cat_*), and the catalytic efficiency (*k_cat_*/K_M_) were determined with GraphPad Prism version 8.

#### 3.5.2. Gas Chromatography-Mass Spectrometry

Activity was determined using 0.05–0.5 mg mL^−1^ purified protein, a NADPH-regenerating system (containing NADPH, Glucose-6-phosphate, and Glucose-6-phosphate dehydrogenase), and 0.3 mM progesterone in a 1 mL final reaction volume [6,10,28]. The reaction was stopped with 1 mL dichloromethane after 2 h at 40 °C incubation. Then, 0.3 mM of testosterone was added as an internal standard. Once the organic phase was collected and dried under air pressure, the residue was dissolved in acetone and analyzed further with GC-MS according to Bauer et al. [10].

## 4. Conclusions

The ability to reduce a variety of substrates is seen as an evolutionary advantage, enabling enzymes to play roles in various metabolic pathways. The divergence in activity within similar enzymes is considered to be caused by functional evolution to better adjust to each corresponding environment. As this adaptability is crucial for survival, adjustment such as the activity and substrate specificity of PRISE have to occur without significant changes in the genome. This research study underlines this statement by verifying previously suggested important residues, referred to as hot spots, that are influential for enzymatic activity within PRISEs, particularly in regard to progesterone as a substrate.

The low conversion of progesterone by *Pl*P5βR1 was reversed by substituting L156 and L346 with the corresponding phenylalanine and alanine residues found in *Pmd*P5βR1, respectively. On the other hand, these mutations had no crucial effects on substrate affinity and the conversion rate of smaller substrates, indicating a much more complex situation of substrate binding, conversion, and release. In addition, the influence of the mutations introduced in *Pmd*P5βR1 had a less pronounced effect in the conversion of progesterone, indicating that a highly active enzyme is much less likely to be rendered unfunctional.

## Figures and Tables

**Table 1:** Identity matrix (%) of amino acid sequences of Cluster I PRISEs from different plants. Clustal Omega used to calculate identities [27].

	*A. thaliana*	*C. roseus*	*D. lanata*	*P. lanceolata*	*P. media*	*P. major*
***A. thaliana***	100	73.32	69.59	72.16	70.88	70.88
***C. roseus***	73.32	100	78.04	78.04	77.26	77.00
***D. lanata***	69.59	78.04	100	87.66	86.63	86.89
***P. lanceolata***	72.16	78.04	87.66	100	92.80	93.57
***P. media***	70.88	77.26	86.63	92.80	100	97.43
***P. major***	70.88	77.00	86.89	93.57	97.43	100

**Table 2:** Kinetic constants of recombinant PRISEs from *P. lanceolata* and *P. media* assayed with progesterone, 8-oxogeranial, MVK, or 2-cyclohexen-1-one, including values for recombinant PRISEs from *P. major* and *D. lanata* taken from [17,19]. All experiments were performed with a minimum of three independent repetitions; SD < 25%.

	**r*Pl*P5βR**			**r*Pmd*P5βR**		
	**K_M_** **[µM]**	***k_cat_*** **[s^−1^]**	***k_cat_*/K_M_** **[M^−1^ s^−1^]**	**K_M_** **[µM]**	***k_cat_*** **[s^−1^]**	***k_cat_*/K_M_** **[M^−1^ s^−1^]**
Progesterone	- *	- *	- *	67	0.20	2985
8-Oxogeranial	100	0.11	1113	19	0.23	12,149
MVK	100	0.56	5530	33	0.55	16,621
2-cyclohexen-1-one	66	0.43	6530	46	0.42	9192
	r*Pm*MOR **			r*Dl*P5βR1 ***		
	**K_M_** **[µM]**	***k_cat_*** **[s^−1^]**	***k_cat_*/K_M_** **[M^−1^ s^−1^]**	**K_M_** **[µM]**	***k_cat_*** **[s^−1^]**	***k_cat_*/K_M_** **[M^−1^ s^−1^]**
Progesterone	28	0.06	2100	360	0.02	52.2
8-Oxogeranial	368	0.9	2600	n. d.	n. d.	n. d.
MVK	n. d.	n. d.	n. d.	270	1.37	5074
2-cyclohexen-1-one	n. d.	n. d.	n. d.	n. d.	n. d.	n. d.

* r*Pl*P5βR1 did not convert progesterone efficiently enough to determine kinetic constants; *n* = 3–6; SD < 25%. ** Data taken from [19]; n. d. = not determined. *** Data taken from [17].

**Table 3:** Docking results represented in empirical binding-free energy using models of PRISEs of *P. lanceolata* and *P. media* with progesterone, 2-cyclohexen-1-one, MVK, and 8-oxogeranial as substrates.

	Progesterone	8-Oxogeranial	2-Cyclohexen-1-One	MVK
Template PDB Structure	5MLH	6GSD	6GSD	6GSD
	**Empirical Binding-Free Energy [kcal mol^−1^]**			
*Pl*P5βR1	−5.4	−2.6	−5.3	−3.9
*Pmd*P5βR1	−6.8	−9.2	−5.1	−3.9

**Table 4:** Kinetic constants for mutants of PRISEs from *P. lanceolata* and *P. media* with either progesterone, 8-oxogeranial, MVK, or 2-cyclohexen-1-one as substrates. Mean of 3–6 independent measurements; SD < 25%.

		r*Pl*P5βR1			r*Pmd*P5βR1		
		L156F	L346A	DMT	F156L	A346L	DMT
Progesterone	K_M_ [µM]	451	170	44	128	218	587
	*k_cat_* [s^−1^]	0.616	0.319	0.278	0.357	0.257	0.578
	*k_cat_*/K_M_ [M^−1^s^−1^]	1366	1882	6318	2789	1179	985
8-Oxogeranial	K_M_ [µM]	8	15	10	9	10	7
	*k_cat_* [s^−1^]	0.154	0.304	0.299	0.245	0.293	0.155
	*k_cat_*/K_M_ [M^−1^s^−1^]	18,125	20,138	29,900	27,222	29,033	22,143
MVK	K_M_ [µM]	47	46	216	24	27	26
	*k_cat_* [s^−1^]	1.093	1.634	1.209	0.697	0.372	0.393
	*k_cat_*/K_M_ [M^−1^s^−1^]	23,255	35,521	5597	29,042	13,778	15,115
2-Cyclohexen-1-one	K_M_ [µM]	16	78	104	72	46	26
	*k_cat_* [s^−1^]	0.528	1.633	1.106	0.363	0.374	0.337
	*k_cat_*/K_M_ [M^−1^s^−1^]	33,000	20,936	10,634	5042	8175	12,962

## Data Availability

Data are contained within the article or Supplementary Material.

## Supplementary Materials

The following supporting information can be downloaded at: https://www.mdpi.com/article/10.3390/molecules29235788/s1. Figure S1: Sequence relationship of PRISEs with 1,4-enone reductase demonstrated with the following substrates: O—8-oxogeranial, P—progesterone, E—small 1,4-enones (<C_10_); Figure S2: Chemical synthesis procedure of 8-oxogeranial from geraniol based on Geu-Flores et al. [4]; Figure S3: TLC analysis of geranyl acetate; Figure S4: GC-MS analysis of geranyl acetate; Figure S5: Analysis of 8-oxogeranial; Figure S6: Analysis of 8-oxogeranial; Figure S7: Expression and IMAC purification of recombinant PRISEs from *P. lanceolata* and *P. media*; Figure S8: Analysis of the heterologous expression of recombinant protein via SDS-PAGE (12%); Table S1: Summary of used primers; Table S2: Summary of constructs and mutated codon triplets using different plasmid templates; Table S3: Summary of point mutations in various PRISEs and activity tested for progesterone; Table S4: Details of models created with SWISS-MODEL. Method: Synthesis of 8-oxogeranial. References [4,6,7,12,17,19,29,51,52,53,54,55] are cited in the Supplementary Materials.
