# Supplementary material for: Addressing the Evolution of Cardenolide Formation in Iridoid-Synthesizing Plants: Site-Directed Mutagenesis of PRISEs (Progesterone-5β-Reductase/Iridoid Synthase-like Enzymes) of Plantago Species
Source: Molecules. 2024 Dec 7;29(23):5788. doi: 10.3390/molecules29235788 (PMC11643720; doi:10.3390/molecules29235788)
Supplement: Supplementary file 1 [file molecules-29-05788-s001.zip › molecules-3293165-supplementary.pdf]

Supplementary Materials

Addressing the evolution of cardenolide formation in iridoid-synthesizing plants: Site-directed mutagenesis of PRISEs (progesterone-5β-reductase/iridoid synthase-like enzymes) of Plantago species

Maja Dorfner[1], Jan Klein[2], Katharina Senkleiter[1], Harald Lanig[3], Wolfgang Kreis[1], Jennifer Munkert*

[1] Friedrich-Alexander-Universität Erlangen-Nürnberg, Department of Biology, Staudtstraße 5, 91058 Erlangen, Germany; maja.dorfner@fau.de (M.D.); katharina.senkleiter@fau.de (K.S.); wolfgang.kreis@fau.de (W.K.)
[2] Friedrich-Schiller-Universität Jena, Department of Plant Physiology, Dornburger Str. 159, 07743 Jena, Germany jan.klein@uni-jena.de (J.K.)
[3] Friedrich-Alexander-Universität Erlangen-Nürnberg, Martensstraße 1, National High Performance Computing Center (NHR@FAU), 91058 Erlangen, Germany; harald.lanig@fau.de (H. L.)
* Correspondence: Friedrich-Alexander-Universität Erlangen-Nürnberg, Pharmaceutical Biology, Department of Biology, Staudtstraße 5, 91058 Erlangen, Germany; jennifer.munkert@fau.de (J. M.); Tel.: +49 9131 8528251

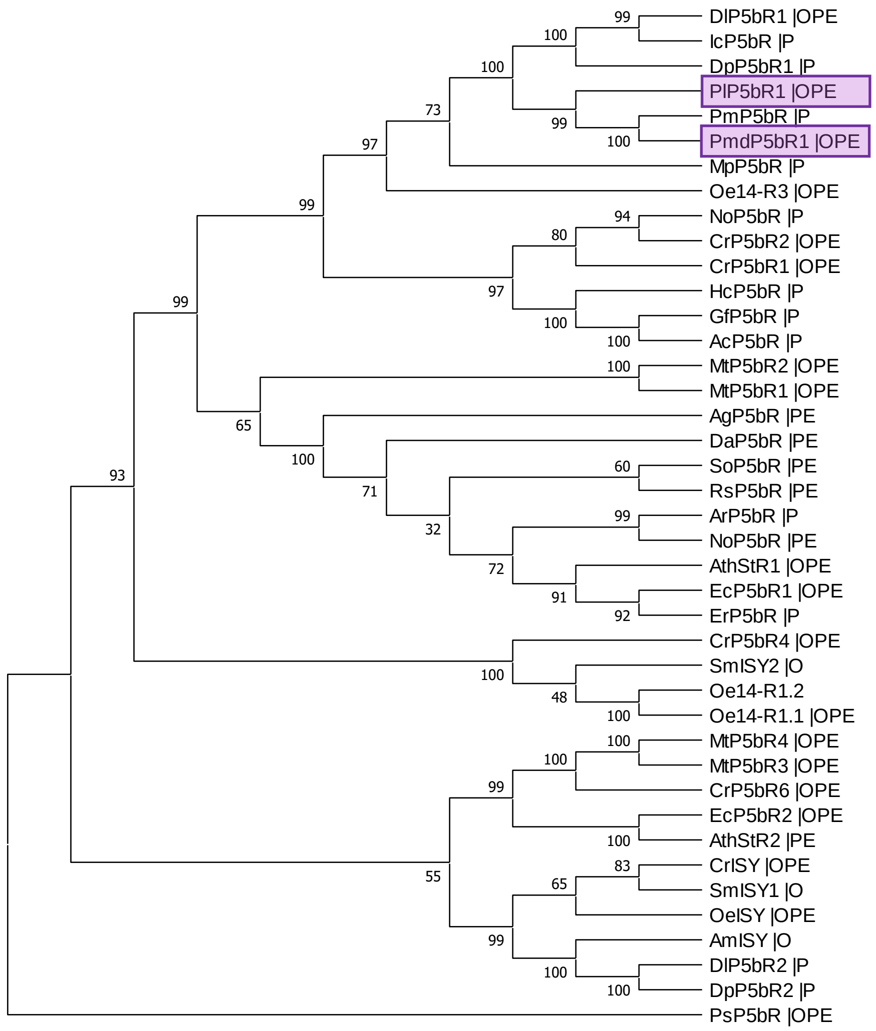

Figure S1. Sequence relationship of PRISEs with 1,4-enone reductase demonstrated with the following substrates: O – 8-oxogeranial, P - progesterone, E - small 1,4-enones (< C_{10}). The evolutionary history was inferred using the Neighbor-Joining method [51]. The optimal tree with the sum of branch length = 5,05183121 is shown. The percentage of replicate trees in which the associated taxa clustered together in the bootstrap test (1000 replicates) are shown next to the branches [52]. The evolutionary distances were computed using the Poisson correction method [53] and are in the units of the number of amino acid substitutions per site. This analysis involved 41 amino acid sequences and the tree is roouted to *Pichea sitchensis* P5bR. All ambiguous positions were removed for each sequence pair (pairwise deletion option). There were a total of 429 positions in the final dataset. Evolutionary analyses were conducted in MEGA X [54]. All positions containing gaps and missing data were eliminated. There were a total of 342 positions in the final dataset. *Digitalis lanata* P5bR1 (AAS76634.1); *Isoplexis canariensis* P5bR (ABB36651.1); *Digitalis purpurea* P5bR1 (AAS93805.1); *Plantago major* P5bR (ADG56541.1); *Plantago media* (WKF48833.1; purple marked); *Plantago lanceolata* (WKF48834.1; purple marked*); Mentha x piperita* P5bR (ADG46022.1); *Nerium oleander* P5bR (ADG56540.1); *Catharanthus roseus* P5bR2 (AIW09144.1); *Catharanthus roseus* P5bR1 (AIW09143.1); *Hoya camosa* P5bR (ADG56539.1); *Gomphocarpus fruticosus* P5bR (ADG56546.1); *Asclepias curassavica* P5bR (ADG56538.1); *Medicago truncatula* P5bR2 (AIW09150.1); *Medicago truncatula* P5bR1 (AIW09149.1); *Raphanus sativus* P5bR (AFZ41789.1); *Draba aizoides* P5bR (AFN22088.1); *Sisymbrium officinale* P5bR (AFZ41796.1); *Amoracia rusticana* P5bR (AEX31541.1); *Nasturtium officinale* P5bR (AFZ42259.1); *Aethionema grandiflora* P5bR (AFZ42259.1); *Arabidopsis thaliana* At4g24220 StR1 (ABU55811.1); *Erysimum crepidifolium* P5bR1 (ADG56544.1); *Erysimum rhaeticum* P5bR (ADG56545.1); *Catharanthus roseus* P5bR4 (AIW09146.1); *Digitalis lanata* P5bR2 (ADL28122.1); *Digitalis purpurea* P5bR2 (ACZ66261.1); *Anthirrhinum majus* ISY (ASM61954.1); *Catharanthus roseus* P5bR5 (AIW09147.1); *Olea europaea* subsp. *europaea* OeISY (ALV83438.1); *Olea europaea* subsp. *europaea* Oe1,4-R1.1 (KT954039), *Olea europaea* subsp. *europaea* Oe1,4-R3 (KT954042) *Picea sitchensis* P5bR (ABK24388.1); *Erysimum crepidifolium* P5bR2 (AGT29343.1); *Arabidopsis thaliana* At5g58750 StR2 (ABL66794.1); *Catharanthus roseus* P5bR6 (AIW09148.1); *Medicago truncatula* P5bR4 (AIW09152.1); *Medicago truncatula* P5bR3 (AIW09151.1); Swertia *mussotii* ISY1 (MF044036); *Swertia mussotii* ISY2 (MF044037).

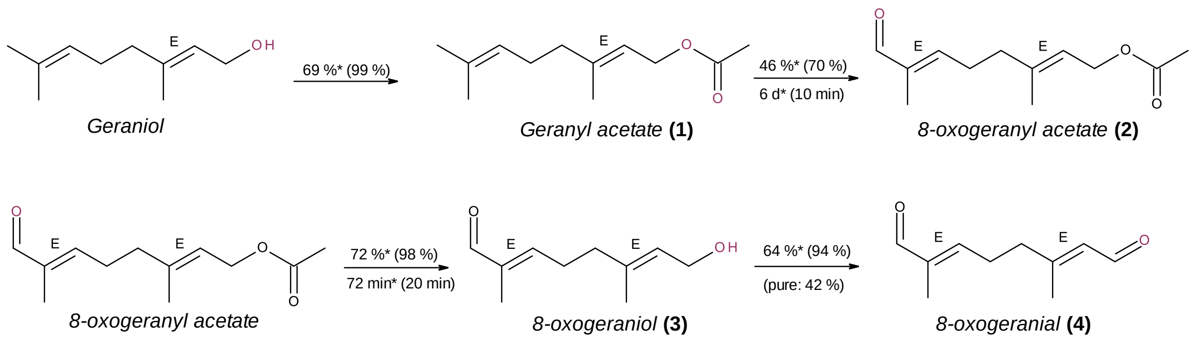

Figure S2. Chemical synthesis procedure of 8-oxogeranial from geraniol based on Geu-Flores *et al.* [4] a) Synthesis of **(1)** – Geraniol, acetic anhydride, DMAP, pyridine, 40 min reaction time. Synthesis of **(2)** – (1), SeO2, tBuOOH, DCM, 6 d. Synthesis of **(3)** – (2), K_2CO_3, MeOH/ H_2O, 72 min reaction time. Synthesis of **(4)** – (3), oxalyl chloride, DMSO, DCM, triethyl amine, 30 min reaction time. Total yield of 8-oxogeranial 14.6 %. Brackets demonstrated the yield after optimization. Optimization strategies resulted in an overall yield of 30 % of 8-oxgeranial.

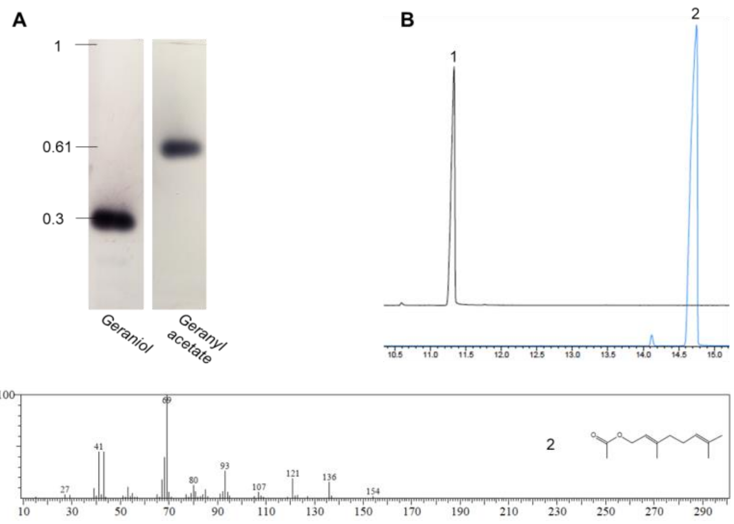

Figure S3. TLC analysis of the reaction product geranyl acetate (Rf = 0.61) formed from geraniol (Rf = 0.3). B) GC-MS analysis of the substrate 1 (geraniol, tR = 11.3 min) and the product 2 (geranyl acetate, tR = 14.6 min).

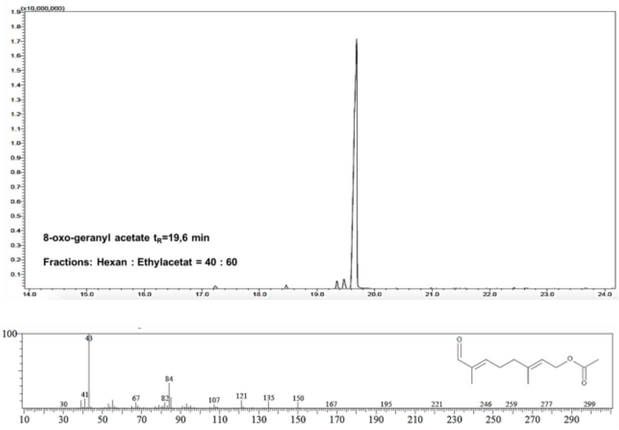

Figure S4. GC-MS analysis of the substrate 1 (geraniol, tR = 11.3 min) and the product 2 (geranyl acetate, tR = 14.6 min).

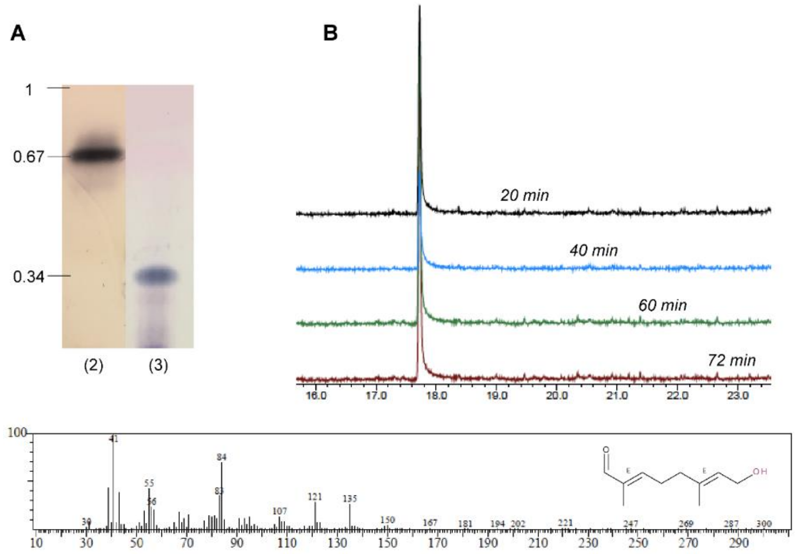

Figure S5. Analysis of 8-oxogeraniol: **A** TLC analysis of substrate (2) (8-oxo-geranyl acetate, Rf = 0.67) and the reaction product (3) (8-oxogeraniol, Rf = 0.34). **B** GC-MS analysis of the reaction product (8-oxogeraniol, tR = 17.7 min) after 20 min, 40 min, 60 min and 72 min.

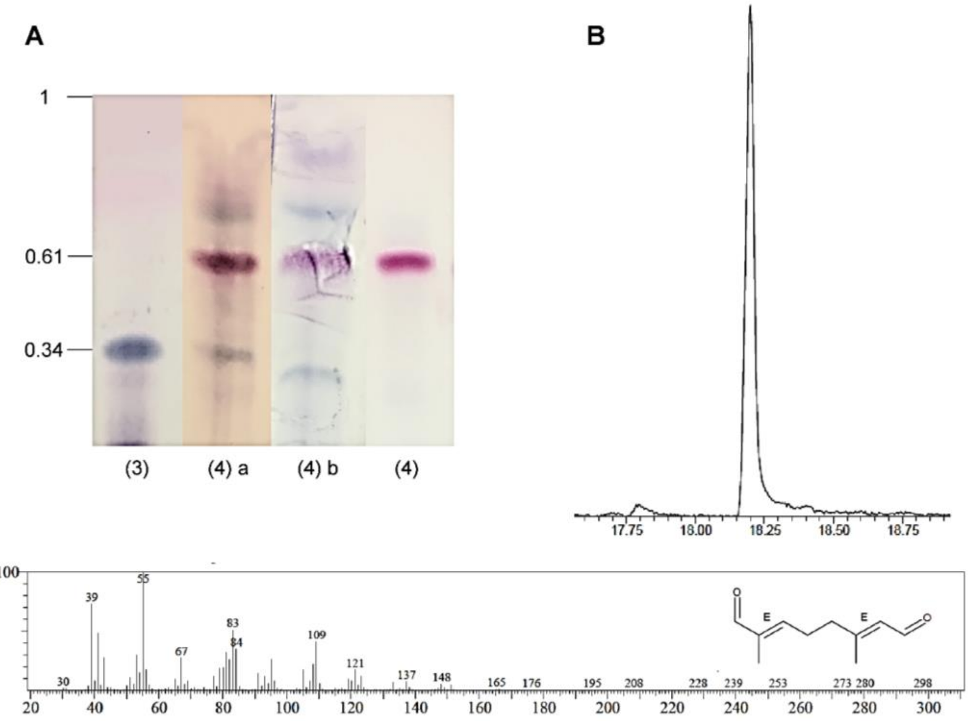

Figure S6. Analysis of 8-oxogeranial: **A** TLC analysis of substrate (3) (8-oxogeraniol, Rf = 0.34) and the purified reaction product (4) (8-oxogeranial, Rf = 0.61). (4) a – reaction product after the filtration, (4) b – reaction product before purification. **B** GC-MS analysis of the reaction product (4) (8-oxogeranial, tR = 18.2 min).

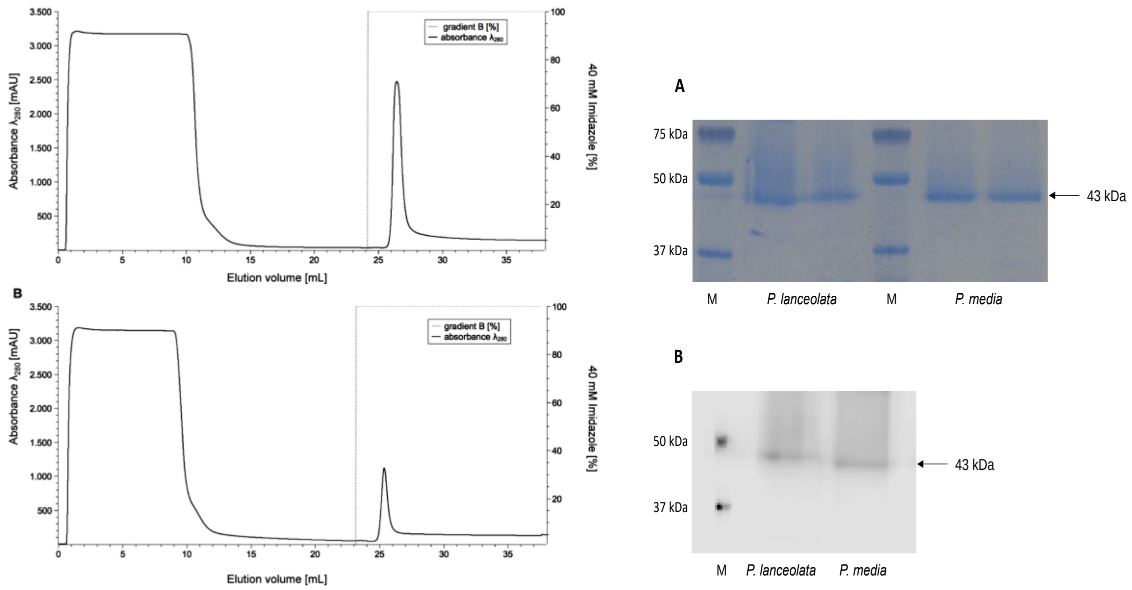

Figure S7. Expression and IMAC purification of recombinant PRISEs from *P. lanceolata* and *P. media.* a) Elution profile after the IMAC purification for the two recombinant PRISEs either from *P. lanceolata* (upper chromatogram) or *P. media* (lower one). b) (A) Expression of recombinant proteins in *E. coli* analyzed on SDS-PAGE (12 %). M – Marker and Western Blot analysis with specific anti-P5βR polyclonal antibody [17] identified a specific protein band at 43 kDa (M – Marker: Precision Protein StandardsTM, BioRAD).

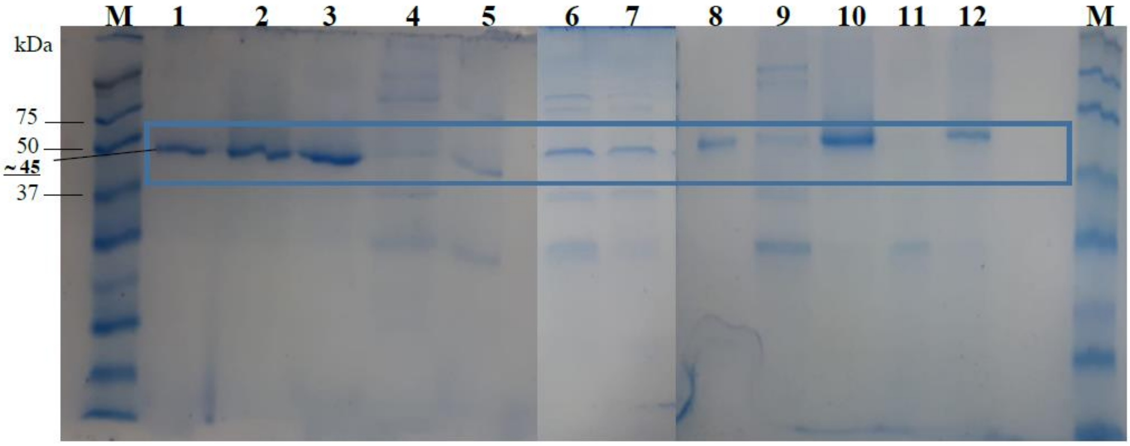

Figure S8. Analysis of heterologous expression of recombinant protein via SDS-PAGE (12%). M-Marker Precision Protein StandardsTM, BioRAD. *Pl*P5βR1: **1**- L156F, **2** - V347I, **3** - L346A, **4** -L346A_V347I, **5**- L156F_L346A_V347I, **6** - L156F_L346A. *Pmd*P5βR1: **7**-L347F_L346A, **8** – F156L, **9** - I347V, **10** - L346A, **11** – A346L_I347V, **12**- F156L_A346L_I347V.

Table S1. Summary of used primers.

Name	Sequence 5′ – 3′
RF_pDEST17_Plantago_for	TCGTACTACCATCACCATCACCATCAC ATGAGYTGGTGGTGGGCT
RF_pDEST17_Plantago_rev	GCCCCAAGGGGTTATGCTAGTTA TCAAGGAACAATCTTGTAAGCCT
*Pl*P5βR1_L156F	G TTT GAG TTA TT**t** GGG AAG ATT GAA TC
*Pl*P5βR1_L346A	G TTT AGT GAT **gc**T ATA CTT GGA TTT CCA TGT C
*Pl*P5βR1_V347I	T AGT GAT CTT **gTg** CTT GGA TTT CCA TGT C
*Pl*P5βR1_L346A_V347I	GG TGG TTT AGT GAT **gc**T **gTg** CTT GGA TTT CCA TGT C
*Pmd*P5βR1_F156L	G TTT GAG TTG TT**a** GGG AAG ATT G
*Pmd*P5βR1_A346L	G TTT AGT GAT **ct**T GTG CTT GAT TAT CCA TG
*Pmd*P5βR1_I347V	T AGT GAT GCT **aTa** CTT GAT TAT CCA TGT CC
*Pmd*P5βR1_A346L_I347V	TGG TGG TTT AGT GAT **ct**T **aTa** CTT GAT TAT CCA TGT

Table S2. Summary of constructs and mutated codon triplets using different plasmid templates.

	Mutation	Codon (AS)	Mutated Codon (AS)	Template
*Pl*P5βR1	L156F	TTA (L)	TTT(F)	Wild type
	L346A	CTT (L)	GCT (A)	Wild type
	V347I	ATA (I)	GTG (V)	Wild type
	L156F_L346A	TTA (L)	TTT (F)	Wild type
	L156F_V347I	ATA (I)	GTG (V)	Wild type
	L156F_L346A_V347I	CTT_GTG (L_V)	GCT_GTG (A_I)	Mutant L156F
*Pmd*P5βR1	F156L	TTT (F)	TTA (L)	Wild type
	A346L	GCT (A)	CTT (L)	Wild type
	I347V	GTG (V)	ATA (I)	Wild type
	F156L_A346L	GCT (A)	CTT (L)	Wild type
	F156L_I347V	GTG (V)	ATA (I)	Wild type
	F156L_A346L_I347V	GCT_GTG (A_I)	CTT_ATA (L_V)	Mutant F156L

Table S3. Summary of point mutations in various PRISEs and activity tested for progesterone.

PRISE origin	Mutant	Activity compared to the wildtype PRISE	Location of the position auf the mutation	Source
Digitalis lanata	T65P	=	Near motif II	[29]
	W106A	- (49%)	Motif IV	[6]
	R146T	=	Near motif IV	[29]
	K147A	- (13%)	Catalytic residue	[6]
	M150L	=	Motif V	[29]
	Y156V	+	Near periphery	[6]
	L182Q	=	Motif VI	[29]
	D181T_L182Q	-	Motif VI	[29]

	G204N	-	Near Motif VII	[29]
	N205L	+	Substrate binding site	[7]
	N205M	+	Substrate binding site	[29]
	N205A	+	Substrate binding site	[29]
	N205M_Y156V	+	Substrate binding site	[29]
	M215A	- (50%)	Substrate binding site	[6]
	S248M	0	Near binding pocket	[29]
	Y302F	=	Near periphery	[29]
	F343A	- (17%)	Near periphery	[6]
	C352	-	Substrate binding site	[29]
	F353L	=	Substrate binding site	[7]
	F353M	=	Substrate binding site	[29]
	F353P	=	Substrate binding site	[29]
	Rc	=	N-terminal	[12]
	Rcn-10	=	N-terminal	[12]
	Rcn-13	=	N-terminal	[12]
	Rcn-20	=	N-terminal	[12]
	GST	=	N-terminal	[12]
	GSTr	=	N-terminal	[12]
Arabidopsis thaliana	F153A	=	Near periphery	[6]
	M204L	+	Near Motif VII	[7]
	F342A	+	Near periphery	[6]
	F153A_F342A	+	Near periphery	[6]
	M352L	+	Binding site	[7]
	F153A_F342A_V156F_V345F	+	Near periphery	[6]
Plantago major	V150M	+	Motif V	[19]
	V150M / I156Y	+	Motif V/ Near periphery	[19]
	A346V / I350N	=	Near periphery	[19]

Table S4. Details of Models Created with SWISS-MODEL.

	Template PDB-ID	GMQE	QMEANDisCo Global	Seq Identity
*Pl*P5βR1	5MLH	0.95	0.88 ± 0.05	93.68%
	6GSD	0.94	0.88 ± 0.05	93.68%
*Pmd*P5βR1	5MLH	0.96	0.89 ± 0.05	97.53%
	6GSD	0.95	0.89 ± 0.05	97.53%

Method: Synthesis of 8-oxogeranial

The synthesized compounds were verified via physicochemical methods:

A. Thin Layer Chromatography (TLC);

B. Gas Chromatography- Mass Spectrometry (GC-MS);

The final product 8-oxogeranial was compared to the reference compound provided by Miettinen et al. [55] as well as to 8-oxogeranial from ChiroBlock GmbH (Wolfen, Germany). Comparing the retention time and corresponding mass spectrum analysis confirmed the identity. Reaction mixtures were purified using column chromatography. The column was loaded with silica gel 60 with a particle size of 230-400 mesh was used as the stationary phase. The ratio of the analyte mixture weight was 1: 300 to the stationary phase weight and 1: 2.5 to the mobile phase.

A. Thin Layer Chromatography

Ready-to-use TLC plates (Silica gel 60G F254) from Merck KGaA, Germany were used as stationary phase. To analyze the samples, they were applied as spots in a solution of 100 µM (1µL in 50 µL DCM). The appropriate mobile phase for each reaction step was chosen to achieve a retardation factor (Rf) of 0.35-0.67: for reaction step 1, a mobile phase of 3:7 (v/v) ethyl acetate/hexane was used; for reaction steps 2 and 3, a mobile phase of 2:8 (v/v) ethyl acetate/dichloromethane was employed; and for reaction step 4, a mobile phase of 8:2 (v/v) ethyl acetate/dichloromethane was utilized. Spots were visualized by immersing the silica plate in p-anisaldehyde stain and developing it at 100 °C on a heating plate.

B. Gas chromatography equipped with mass spectroscopy

The synthesized compounds were confirmed through gas chromatography-mass spectrometry (GC-MS) analysis. DB-5 ms column (30 m x 0.25 µm x 0.25 µm) acted as the stationary phase and helium as the mobile phase with a flow rate of 1.74 mL min^{-1}. The injection temperature of 1 µL sample volume in injection mode split was set to 250 °C. The products were dissolved in 100 µL dichloromethane and transferred into GC vials. The measurements were done with Shimadzu GC-MS-QP2010S. The program started at 60 °C and ran at 5 °C min^{-1} up to 150 °C, a 20 °C min^{-1} gradient up to 240°C, 20 °C min^{-1} up to 290 °C, and 5 min isothermal at 290 °C. For mass spectrometry the ion source temperature was 230 °C, the interface temperature 250 °C and the solvent cut time of 2 minutes.

1.1 Synthesis of geranyl acetate from geraniol

A solution of DMAP (0.72 mg, 5.9 mmol) in anhydrous pyridine (3.7 mL) was prepared and cooled to 0 °C in ice/water bath while stirring. Geraniol (2 g, 13 mmol) was added dropwise to the cooled mixture, followed by the addition of acetic anhydride (3.7 mL, 39 mmol). The flask was covered with its original stopper, sealed with parafilm and the reaction mixture was stirred in a cooling chamber (2 h, 10 °C). Afterwards ice water (60 mL) was added to the mixture, which was then transferred to the separating funnel. The mixture was extracted with hexane (2 x 50 mL). The combined organic phases were washed sequentially with 10 % aqueous HCl (100 mL) and then with 25 % saturated NaHCO3 (aq) (50 mL). The organic layer was dried over $MgSO_4$, and the solvent was removed under reduced pressure at 20 °C to yield geranyl acetate as a clear, colorless liquid with a faint lavender scent. The reaction was monitored with TLC and GC-MS (Figure S3 and S4)

1.2. Synthesis of 8-oxogeranyl acetate from geranyl acetate

SeO_2 (25.7 mg, 0.23 mmol) and tBuOOH (62.6 µL, 0.65 mmol) were combined, followed by the addition of dichloromethane (DCM) (500 µL). The solution was vortexed until thoroughly mixed, after which the synthesized geranyl acetate (109.9 µL, 0.46 mmol) was added. A total of 320 mg of silica was spread evenly in a 10 cm diameter petri dish, and the reaction mixture was distributed uniformly onto the silica.

Once the DCM had evaporated in air, the silica coated with the reaction mixture was microwaved for 20 minutes at 450 W, with manual shaking every two minutes to ensure even heating. After cooling, diethyl ether was added to the mixture, which was then filtered three times (3 x 20 mL). The filtrate was washed with 10% NaOH until the aqueous phase became colorless (4 x 20 mL), and any remaining water in the reaction mixture was removed by drying over $MgSO_4$. The solvent was further evaporated, and the amount of the product formed was determined by GC-MS (Figure S5).

1.2.1. Purification of 8-oxogeranyl acetate by column chromatography

The synthesized product (78 mg) was purified by column chromatography (column length 28 cm, width 4.5 cm, flow rate 10 mL min^{-1}), eluting with a step gradient of hexane and ethyl acetate in 10 mL fractions (hexane ‣ ethyl acetate: 100 ‣ 80 ‣ 70 ‣ 50 ‣ 40 ‣ 20 ‣ 0). In this way, 8-oxogeranyl acetate was isolated at the gradient step of 50:50 to 40:60 (hexane: ethyl acetate). The presence of the product was visualized by TLC and GC-MS.

1.3. Synthesis of 8-oxogeraniol from 8-oxogeranyl acetate

The synthesis of 8-oxogeranyl acetate was performed in accordance with the literature conditions [4].

1.4. Synthesis of 8-oxogeranial from 8-oxogeraniol

The synthesis of 8-oxogeranial was also performed according to Geu-Flores et al. [4] with minor modifications. Oxalyl chloride (40 µL, 0.46 mmol, 1.55 equiv) was dissolved in DCM (500 µL) and cooled to -78 °C while stirring in dry ice in acetone. Dimethyl sulfoxide (40 µL, 0.56 mmol, 1.88 equiv) in DCM (500 µL) was added slowly over two minutes with vigorous stirring and cooling. After 15 minutes, 8-oxogeraniol (55 µL, 0.3 mmol, 1 equiv) in DCM (500 µL) was added dropwise for three minutes and the solution was stirred for an additional 30 minutes. After that, triethylamine (124 µL, 0.89 mmol, 3 equiv) was added drop by drop and the solution was stirred for at least five minutes, before the dry ice was removed. The solution was allowed to return to room temperature (20 °C) while stirring (30 min), during which triethylammonium chloride formed. The mixture was then filtered through a 1 cm MgSO4 plug on a fritted glass Büchner funnel and the filter was washed with 15 % diethyl ether in hexane (3 x 20 mL). The filtrate was swirled and filtered through a 1 cm celite pad which was washed with 15 % diethyl ether in hexane (3 x 10 mL). The collected filtrate was concentrated under reduced pressure to yield a light yellow oily solution.

1.4.1. Purification of 8-oxogeranial by column chromatography

The prepared product (119 mg) was purified by column chromatography (column length 15 cm, width 4.5 cm, flow rate 10 mL/min in 10 mL fractions), eluting with a step gradient of hexane and ethyl acetate (hexane ‣ ethyl acetate: 100 ‣ 70 ‣ 60 ‣ 50 ‣ 40 ‣ 20 ‣ 10 ‣ 0). In this way, 8-oxogeranial was isolated at a gradient step of 40:60 (hexane: ethyl acetate). The isolated product was analyzed by TLC and GC-MS (Figure S6) and compared to the commercially available 8-oxogeranial standard
